# Clinical Variability in Arch Wires: A Preliminary Study Evaluating Mechanical and Surface Characteristics of Two Different Sized Rectangular Stainless Steel Wires

**DOI:** 10.2174/1874120700701010013

**Published:** 2007-08-03

**Authors:** Alessandro Vena, Jason Carey, Hisham Badawi

**Affiliations:** a 4-9 Mechanical Engineering Building, University of Alberta, University of Alberta, Edmonton, Alberta, Canada T6G 2G8; b Department of Mechanical Engineering. University of Alberta, Edmonton, Alberta, Canada T6G 2G8; c Faculty of Medicine and Dentistry, University of Alberta, Edmonton, AB, Canada

**Keywords:** Arch wires, mechanical properties, geometry, consistency.

## Abstract

Experimental characterization of arch wires has been performed in many previous studies; however with the advent of new arch wire materials being introduced, some new experimental methods and characterization are required. Since literature is available for comparison, this paper examines mechanical and physical characteristics of steel arch wires to quantify their variability in engineering terms. Furthermore, the effect of wire size on properties was evaluated using two of the most common wire sizes. Finally, manufacturing consistency was verified by testing samples from different lots.

## INTRODUCTION

Since the beginning of fixed appliance therapy in the early nineteen hundreds, arch wires have become increasingly sophisticated. When compared with stainless steel wires, many newly introduced alloys, such as beta titanium and nickel titanium, have lower stiffness, superior biocompatibility properties and better corrosion resistance [1]. These specialized wires even promise shape memory properties and the possibility of super elastic behavior, which significantly impacts clinical practices. However, even with the advancements provided by these new materials, standard stainless steel arch wires are still the material of choice in many stages of treatment. They provide an attractive combination of stiffness, resilience and formability.

Since arch wires are the main force system in orthodontics, it is important in clinical practice that they deliver appropriate, predictable and repeatable forces during extraction. The main concern with the newer heat activated alloys is that they require a specific phase composition to exhibit shape memory and super elastic properties, which makes them difficult to manufacture consistently [2]. On the other hand, stainless steel has the advantage of being simple to manipulate and has been manufactured for decades. However, clinical practitioners have commented on the variability of arch wire behavior for years. Inconsistent arch wire properties can contribute to unpredictable treatment duration and results.

Orthodontic arch wires have been experimentally tested many times over. In some cases, the objective of the study was to measure the mechanical properties of wires, thus quantifying their mechanical behavior. This characterization eliminates some of the subjectivity involved in choosing the appropriate wires at various stages of treatment. Many other studies have attempted to compare and contrast the properties of different alloys, thus determining the advantages and disadvantages of newer materials [1,3]. However, few articles [4,5,6,7,8,9] have studied the consistency of the mechanical properties of stainless steel wires, especially the consistency along the length of the wire. Furthermore, many authors have shown that the edge bevel of wire has an effect on torque capability [10,11,12], which explains the importance of cross-section shape.

As the main governing body in orthodontics, the American Dental Association (ADA) [13] lists a number of testing methods to ensure that all variables remain constant from one study to another. It states that the mechanical properties of orthodontic arch wires are to be determined by a symmetric three-point bend test. The specimens must have a minimum length of 50 mm, exhibit symmetrical bending in the plane of the specimen thickness and be subjected to a maximum center point deflection rate of 10 mm/min. The test span must be 12 mm, with the knife-edge supports and striker having radii of between 0.05 mm and 0.13 mm. For stainless steel wires, Type 1 wires (linear elastic), the test must be carried out at 23 ± 1°C. Furthermore, the load measurements are to be taken during the unloading or deactivation, as this best represents the forces exerted during clinical practice. A sample size of 10 wires is required by the ADA for each type of wire. Although this testing method may provide practitioners with clinically relevant information on the mechanical behavior of arch wires, it does not provide information on their mechanical properties. These properties could serve as a basis for comparison between different types of wires, which served as the purpose for the following studies.

Krishnan and Kumar [3] have tested Ormco stainless steel 0.017 x 0.025 in wires in tension to obtain the elastic modulus, yield strength and ultimate tensile strength. The following results were found: elastic modulus of 170 ± 20 GPa, 0.2% offset yield strength of 1640 ± 70 MPa and ultimate strength of 2100 ± 40 MPa. Verstrynge, Humbeeck and Willems [7] have also tested Ormco stainless steel 0.017 x 0.025 in wires in tension to obtain the tensional elastic modulus, yield strength and ultimate tensile strength. The following results were found: tensional elastic modulus of 166 ± 1 GPa, 0.2% offset yield strength of 1699 MPa and ultimate strength of 1986 MPa. Rucker and Kusy [8] have tested Ormco stainless steel wires, diameter of 0.012 in, in tension, to find the ultimate tensile strength and yield strength, and in bending, to find the elastic modulus. The following results were found: elastic modulus of 198 GPa, 0.1% offset yield strength of 1720 ± 70 MPa and ultimate strength of 2280 ± 80 MPa.

Stainless steel arch wires are manufactured by way of cold drawing followed by finish rolling to size. These processes enhance the mechanical properties of stainless steel, increasing the yield and ultimate tensile strengths. However, this increase in strength is accompanied by a decrease in formability, which is a very important characteristic for arch wires. Furthermore, the plastic deformation that is produced during the manufacturing process may cause residual stresses in the material, thus making it more brittle in tension and poor in fatigue. By measuring the properties along the length of the wire, we can determine whether or not these adverse effects, inherent to the manufacturing process, have an appreciable impact on the mechanical properties of the wires. The purpose of this study is to quantify the variability of arch wires in engineering terms by measuring the mechanical properties and surface characteristics of stainless steel wires along their length. The tensile, torsional, flexural, and surface properties as well as cross sections of Ormco stainless steel 0.019 x 0.025 in and 0.017 x 0.025 in wires are studied.

## MATERIAL AND METHODS

The rectangular stainless steel orthodontic arch wires investigated were SDS Ormco (Glendora, California), sizes 0.019 x 0.025 in (Group I) and 0.017 x 0.025 in (Group II).

These specimens were tested for modulus of elasticity (E), yield strength (S_y_), ultimate tensile strength (UTS), modulus of resilience (U_r_), elastic shear modulus (G), flexural rigidity (EI), cross-sectional area and shape and surface characteristics.

### Tensile Properties

**Fig. (1):**
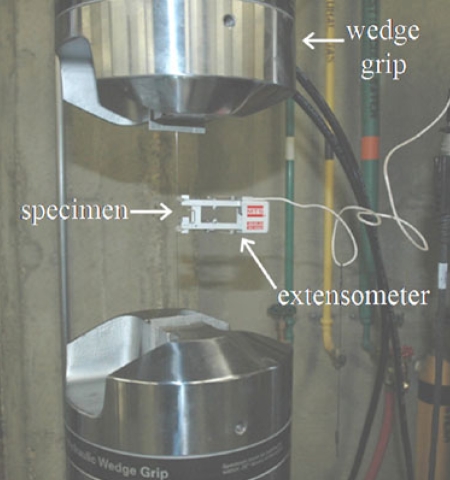
Tensile test using MTS 810 Material Testing System and MTS Extensometer.

A standard tensile test was performed on Groups I and II in an MTS 810 Material Testing System (MTS Corporation, Minneapolis, Minnesota) using MTS 647 Hydraulic Wedge Grips (Fig. **1**). The specimens were randomly taken from 5 different lots and 10 specimens were tested for each group. The gauge length of each specimen was set to 100mm. The crosshead speed was set to 1mm/min and the load cell measured the axial load at a frequency of 100 Hz. The axial strain on the wire was measured using an MTS Extensometer. Model No 634.12E-24. The axial load and axial strain data obtained were plotted as stress-strain curves. The slope of the linear portion of the curves yielded E, as given by the following formula

(1) $$E=\frac{\sigma }{\epsilon }$$

where *σ* is the applied stress and *ε* is the axial strain.

S_y_ was measured at a 0.2% strain offset; and, UTS was found from the maximum axial stress. The modulus of resilience, U_r_, is the area under the stress-strain curve up to yielding. It represents the amount of recoverable energy during unloading. This energy translates into applied forces on the bracket, which are the result of the wire returning to its under formed state. It is calculated from

(2) $$U_{r}=\frac{1}{2}\sigma_{y}\epsilon_{y}=\frac{\sigma_{y}^{2}}{2E}$$

where *σ_y_* is the yield stress and *ε_y_* the yield strain.

### Elastic Shear Modulus

**Fig. (2):**
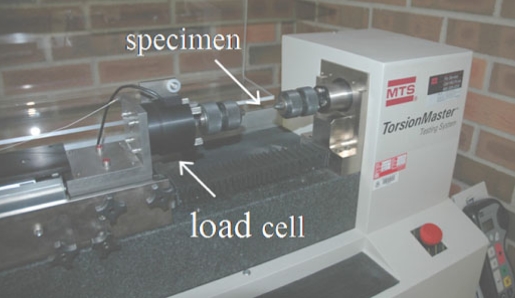
Torsion test using MTS Torsion Master Testing System Model No. 27.000135.

A standard torsion test was performed on Groups I and II in an MTS Torsion Master Testing System Model No. 27.000135 (Fig. **2**). The samples were randomly taken from 5 different lots and each sample (wire) was then divided into 4 specimens. A total of 10 samples were tested per group. The gauge length of each specimen was 55 mm. The wire was subjected to a constant angular rotation of 0.1 rad/s and the load cell read the torque applied to the wire. When plotting the torque as a function of the angle of twist, the linear portion of the graph yields the torsional stiffness, *k_t_*, of the specimen. The elastic shear modulus, G, could then be calculated with the following equation

(3) $$G=\frac{T}{\phi }\cdot \frac{L}{{\beta \mathrm{bt}}^{3}}=k_{t}\cdot \frac{L}{{\beta \mathrm{bt}}^{3}}$$

where *L* is the length of the specimen, *b* is the width of the specimen, *t* is the thickness of the specimen and *β* is a function of *b* and *t* [14].

### Flexural Rigidity

**Fig. (3):**
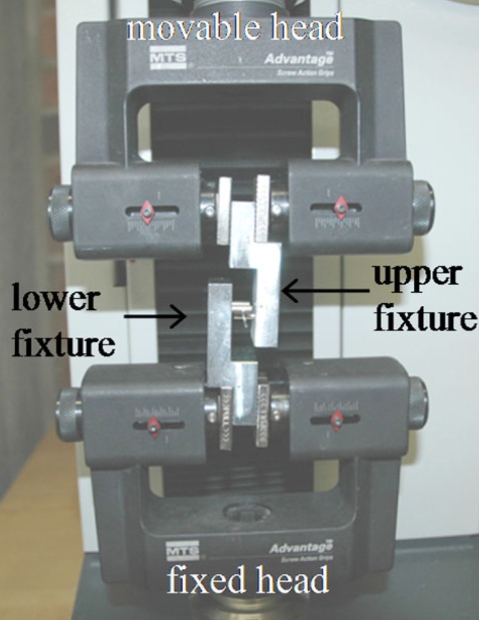
Three-Point bend test using MTS Synergy 400 Tensile Tester.

**Fig. (4):**
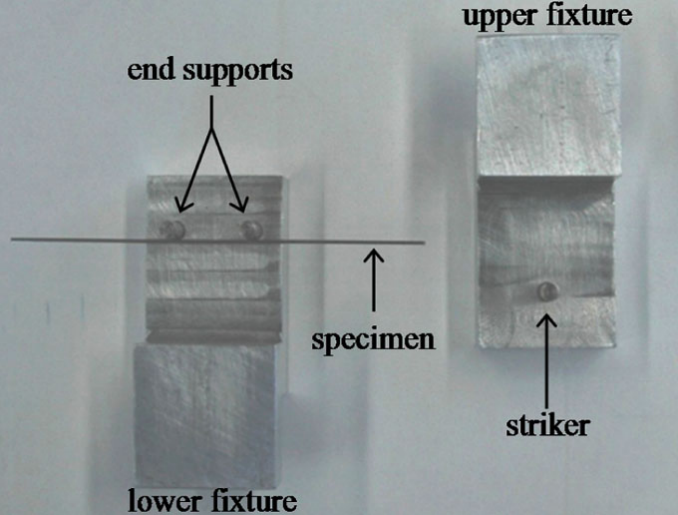
Three-Point bend test fixtures.

The load deflection characteristics of Groups I and II were determined by way of a standard three-point bend test (Fig. **3**). An MTS Synergy 400 Model No. 27.00094 tensile tester was used with custom made fixtures (Fig. **4**). The lower fixture consisted of two round aluminum bars of 1/8″ in diameter acting as end supports. These have a larger radius than described by the ADA, but were used to avoid slippage and knife-edge caused frictions. The end supports were placed 14 mm apart, not 12 mm as specified by ADA, for this distance is currently used as the average distance between the labial centers of a lower incisor and first premolar [15]. The lower fixture was secured onto the fixed head of the tensile tester. The upper fixture consisted of one round aluminum bar of 1/8″ in diameter acting as a striker and, when attached to the upper movable head of the tensile tester, would strike at the midpoint of the arch wire. This would replicate an inter-bracket distance of 7 mm. The midpoint of the wire was subjected to a load resulting in a 1mm deflection, at a cross head speed of 1mm/min, and then unloaded to its original state. The samples were randomly taken from 5 different lots and each sample (wire) was then divided into 5 specimens. A total of 10 samples were tested per group. When plotting the applied load as a function of the midpoint deflection, the flexural rigidity, EI, can be calculated using the following equation

(4) $$EI=\frac{P}{\Delta }\cdot \frac{L^{3}}{48}$$

where *P* is the applied load, *Δ* is the center point deflection, $\frac{P}{\Delta }$ is the slope of the linear portion of the graph and *L* is the length of the specimen.

### Cross-Sectional Area and Shape

**Fig. (5):**
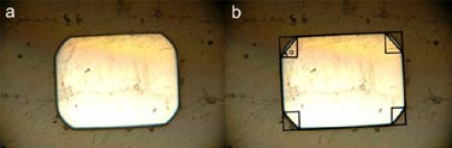
Magnified cross-section photograph, Magnification 60x, Group I, Specimen; (**a**) Magnified cross-section, (**b**) Bevel angle measurement.

To better observe the cross-sectional area and edge bevel along the wires, 10 samples were randomly selected from 5 different lots and each sample (wire) was cut into 5 specimens, both for Groups I and II. The specimens were then placed vertically in a cup containing a two-part resin. Once the resin hardened, the upper surface of the cup was polished to reveal the cross-sections. The surface was sanded in 4 steps using, 80, 240, 400 and 600 grits, followed by polishing with loose abrasives (Aluminium oxide abrasive grinding powder 5.0 μm). Using a metallurgic microscope (Zeiss Camera Microscope Ultraphot) and digital camera (Nikon D70), the cross-sections were individually photographed at different magnifications (Fig. **5a**). The cross-sectional area was calculated using ImageJ software (developed at the National Institutes of Health, Washington, DC. USA), where the contour of the cross-section was outlined using a series of mapped points. Initially, edge bevel was to be calculated by matching the radius of curvature to circular preforms; however this method proved very inconsistent and inaccurate since edges are not circular. It was deemed more important to assess the bevel angle which could be determined by trigonometry (Fig. **5b**) as it has an affect on the effective torque applied by the wire. The bevel angles were averaged at all four corners of the specimen’s cross-section, then average for all five specimens of that sample, yielding the inter-sample average bevel angle.

### Surface Characteristics

The surface characteristics of the wires were studied by using a field emission scanning electron microscope (FESEM; JE0L 6301F). The specimens were mounted onto studs and placed in the SEM vacuum chamber. Their surfaces were then scanned and viewed at different magnifications. To study the effects of the previous mechanical tests on the surface of the wires, one as-received specimen and one post-specimen from every test was studied, both for Groups I and II.

### Statistical Analysis

Descriptive statistics, including means and standard deviations, were calculated for all mechanical properties, cross-section dimensions and edge bevel angles. Analysis of variance was carried out by conducting a Student’s t-Test and statistical significance was found when P < 0.05. This procedure was applied to all samples within a group, to determine significance of samples when compared to their entire group, and was applied to compare Groups I and II, to determine if there was significance between groups.

## RESULTS

### Tensile Properties

**Fig. (6):**
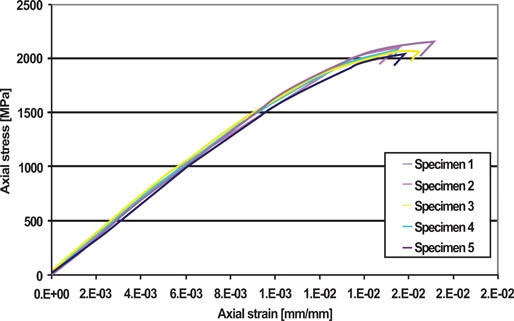
Tension test results; Axial stress *vs* Axial strain; Group I.

The load deflection curves obtained by tensile testing were plotted as stress-strain curves for each specimen (Fig. **6**). The mechanical properties obtained from these curves are summarized in Table **1**. First, Groups I and II display a very consistent linear portion of their respective curves, which is evident by the small inter-group standard deviation in E. Secondly, S_y_ and UTS are also very consistent, showing little inter-group variation. The results for Group II can be compared with those found by Krishnan and Kumar [3] and Verstrynge, Humbeeck and Willems [7] for Ormco stainless steel 0.017 x 0.025 in. The percent error for E and UTS range from 1.0 to 1.4% and 0.1 to 5.6%, respectively. This study shows comparable standard deviation for E, at 4% of mean value (compared to 0.6 to 11.8%), and standard deviation of UTS also at 4% of mean value (compared to 1.9%). However, the yield strength is much higher in this study; there is a 19 to 24% error difference. The modulus of resilience also shows very small inter-sample standard deviation. Furthermore, the standard deviation in the modulus of resilience for Group I and Group II are 8% and 5%, respectively. Group II does display a slightly larger U_r_, which is due to a slightly higher S_y_, but no statistical significance was found (P < 0.05). Thirdly, it can be said that fracture does not occur at the same stress-strain conditions for samples in Group I. Some specimens fracture slightly after attaining UTS, while others undergo significant plastic deformation. However, it is experimentally difficult to predict the location of fracture of a specimen that does not have a “dog bone” shape. Since the main weakness of the specimen occurs in the grips, due to stress-concentration, fracture will become very sensitive to testing conditions.

### Elastic Shear Modulus

**Fig. (7):**
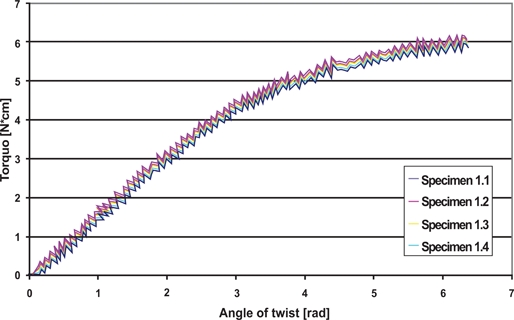
Torsion test results Group I, Sample 1; Torque *vs* Angle of twist.

The measurements from the torsion test were plotted, yielding the applied torque as a function of angle of twist (Fig. **7**). From the linear portion of the graph, the torsional stiffness was found and G calculated. The results are shown in Table **2**. The linear portion of the curve, up to an angle of twist of approximately 4 radians, has a very low standard deviation within each sample. This shows that there is very little change in the torsional properties along the wires. The results also show that 6 samples, within each group, showed statistically significant differences with their respective group. This shows that the torsional properties did vary from sample to sample.

### Flexural Rigidity

**Fig. (8):**
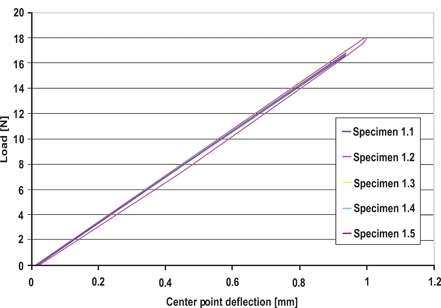
Three-Point bend test results Group I, Sample 1; Load *vs* Center point deflection.

The data obtained from the three-point bend test was plotted as load versus midpoint deflection (Fig. **8**). The slope of the loading curve yields the stiffness, from which EI can be calculated. Results are presented in Table **3**. Groups I and II display very small standard deviations for EI, which is evident by superimposing the loading curves. As expected, the EI of Group II is noticeably smaller than Group I (P << 0.05), as this is a material and geometric property and the moment of inertia (I) is proportional to the cross-section dimensions.

### Cross-Sectional Area and Shape

The cross-sectional area of the wire sections was calculated by mapping the contour with a series of points in ImageJ and then measuring the enclosed area. The results are shown in Table **4**. Groups I and II exhibit a very low inter-sample and inter-group standard deviation, showing that the cross-sectional area remains relatively constant along the length of the sample and within the same group. Sample 7 was not included in the inter-group calculations for Group II. This outlier could possibly belong to Group I, which would explain its increased area. As expected, statistical significance was found between groups. Furthermore, the average bevel angles of the wire sections are shown in Table **5**. Groups I and II exhibit moderate inter-group standard deviation, although both groups display significant inter-sample standard deviation. This shows that for some samples the bevel angle does vary along the length of the wire, as well as within the cross-section, as evident by visual inspection (see Fig. **5**).

### Surface Characteristics

Fig. (**9**) shows the scanning electron micrographs of Group I in 500X magnifications. The as-received arch wires exhibited a rather smooth surface with some vertical cracks and virtually no pores. There was also evidence of dark spots scattered along the surface, with no apparent orientation or repeating pattern, which could indicate manufacturing problems. The post-tensile specimen exhibited a very rough surface, with several large deep cracks in the direction of axial loading. The width of these cracks is approximately 50X that of the as-received specimen, indicating that the surface cracks propagated in tension. The post-torsion specimen exhibited a rougher surface than the as-received specimen, showing longer and more frequent surface cracks. The width of these cracks is approximately 10X that of the as-received wires. The cracks, as in the case of the post-tensile specimen, are also along the longitudinal axis of the specimen. The post-three-point bend specimen displayed the smoothest surface of all post-specimens. It displayed surface cracks roughly 5X that of the as-received specimen, but still maintained a similar overall roughness.

Fig. (**9**) also shows the scanning electron micrographs of Group II in 500X magnifications. The as-received arch wires displayed a smooth surface similar to that of Group I, with few cracks and virtually no pores. However, the post-tensile specimen displayed a surface finish with very few imperfections, where the width of the surface cracks is approximately 10X that of the as-received wires. The post-torsion specimen displayed a slightly rougher surface than the as-received specimen, with more frequent surface cracks and pores. Again, the width of the surface cracks is approximately 10X that of the as-received wires. The post-three-point bend specimen displayed a surface with several dark spots, but displaying little difference with the as-received wires.

Fig. (**10**) shows the scanning electron micrographs of the fracture surface of Group I. Figure (a) displays the entire fracture surface of the specimen, which shows a slight amount of necking, indicative of a moderate ductile fracture. Figure (b) examines the central fibrous region of the fracture surface, showing the random size grains. These spherical dimples formed during the fracture process, each representing one half of a microvoid. This confirms that the material was at least moderately ductile, showing no signs of brittle fracture. Fig. (**10**) shows the scanning electron micrographs of the fracture surface of Group II, which displays the same properties as Group I, with a slight amount of necking and a fibrous inner surface, which is completely covered with dimples.

## DISCUSSION

The main objective of the study was to examine the variability of stainless steel arch wires with respect to mechanical properties, cross-section dimensions and overall surface finish. Two different sized rectangular stainless steel wires were chosen to be studied, Groups I and II. The findings for each wire size are discussed and then compared.

### Group I: Ormco Stainless Steel .019x.025

A tensile test is the most common mechanical stress-strain test, where a specimen is axially loaded in tension, under a constant strain rate, until fracture. The resulting stress-strain curve yields the elastic and plastic behavior of the material. Fig. (**6**) illustrates the tensile behavior of Group I. The S_y_ and UTS show that the resulting arch wires have very high strength; approximately four times typical values of annealed 302 stainless steel [16]. The strain hardening that occurs during the manufacturing process gives the wires this increase in strength and, by increasing the yield strength, increases the elastic region of the stress-strain curve. As a result, the wire requires higher stresses to produce a plastic deformation, indicating that a larger amount of recoverable energy can be stored during loading. This is evident by the large value of U_r_, which shows the high spring back of the wires. However, the resultant material becomes more brittle, and as a consequence cannot withstand as much plastic deformation before fracture. Therefore, the manufacturing process increases the spring back of the wires but decreases their formability.

The torsional characteristics of arch wires are crucial for they must often be twisted to properly engage the bracket. Torque transmission is therefore very important to practitioners, and the elastic torsional behavior must be predictable. The torsion test performed showed that G was consist, inter-sample, with small standard deviations with the exception of sample 10. However, 6 samples showed statistically significant variability when compared to the entire group. This indicates that the wires have reliable torsional behavior along their length, but display slight variability between wires.

The most clinically relevant approach to represent the forces involved during extraction is by way of a three-point bend test. By controlling the amount of midpoint deflection given to the wire, it is possible to find its flexural rigidity, EI, which is both a material and geometric property. This best describes the restriction of the wires to bending during activation of a bracket. The three-point bend test performed showed an EI of approximately 1 x 10^-3^ Nm^2^, which is smaller than calculated for a rectangular cross-section of the listed dimensions. The wire is slightly more flexible due to the edge bevel, which decreases the moment of inertia in the direction of bending. The results show very little inter-sample and inter-group variations.

The cross-section area and bevel angle play an important role in the interaction between the arch wire and bracket, the latter having a significant effect on torque transmission. The cross-section area results show very little inter-group and inter-sample variability, although the bevel angle results display larger inter-sample variability. These results may be a consequence of the cold rolling to size performed during the manufacturing process. Although cold rolling is an effective procedure in sizing the thickness and width of the cross-section, it causes considerable residual stresses which could cause local deformation. These local deformations could contribute to the variability in the bevel angle, resulting in a somewhat inconsistent cross-section shape.

The surface quality of arch wires is very important, as it determines corrosion resistance, biocompatibility and frictions characteristics, the latter having larges effects on the force transmission between arch wire and bracket. Scanning electron microscopy of the as-received specimen exhibited a rather smooth surface with some vertical cracks and virtually no pores; this low roughness is advantageous for it reduces friction. The post-tensile and post-torsion specimens displayed very rough surfaces, with large deep cracks and subsequent voids. These irregularities will act as stress raisers, which will weaken the material and make it more susceptible to fracture. In clinical practice, wires are constantly tightened and adjusted during treatment, which could result in plastic deformations. Consequently, the surface roughness would increase and would result in an increase in frictional losses. The post-three-point bend specimen showed very little change in surface characteristics since there was little plastic deformation.

Another effective way to characterize a material is to identify its fracture mode. To do so, the fracture surface of the post-tensile specimen was examined. The scanning electron microscopy is shown in Fig. (**10**). Shown is an appreciably reduced cross-section, due to necking, and a fibrous inner fracture surface completely formed of spherical dimples. Although the wires did not show a very large percent elongation during the tensile test, the fracture surface is at least typical of a moderately ductile material. This shows that the material does not fracture immediately after reaching its yield stress, but does show a slight amount of plastic deformation. In clinical practice, this means that the wires will display plastic deformation before they fail.

### Group II: Ormco Stainless Steel .017x.025

The tensile stress-strain results of Group II are shown in Table **1**. The values of E and UTS are very similar to those found by Krishnan and Kumar [3] and Verstrynge, Humbeeck and Willems [7], with the exception of S_y_ which was higher in this study. The results show a high strength material with a large amount of springback, like Group I, but with a slightly more brittle nature. The arch wires reached their UTS shortly before fracture, with very little plastic deformation. This confirms that the strain hardening resulting from the manufacturing process leaves the wires quite brittle, which significantly limits their formability. Perhaps the smaller wires required more cold rolling to size, which could explain why they are more brittle than Group I.

The torsional characteristics of these wires are very similar to Group I, with similar values of G and comparable standard deviations. Because of the smaller cross-sectional thickness of Group II, less torque needs to be applied to the wires to produce the desired angle of twist. Therefore, it would be easier to engage these wires into a poorly aligned bracket.

The three-point bend test performed showed an EI of approximately 0.77 x 10^-3^ Nm^2^, which is about 23 percent less than that of Group I. This means that the smaller cross-section dimensions result in a smaller moment of inertia and reduce the restriction to bending of the wire. This would imply that less force is needed to deflect the wire and slot into the bracket.

The cross-sectional characteristics of the wires are very similar to those of Group I, since the cross-section area results show very little inter-group and inter-sample variability, although the bevel angle results display noticeable inter-sample variability. Again, it is quite possible that this variability in bevel angle is a result of the residual stresses induced during cold-rolling, causing inconsistent local deformation.

The scanning electron microscopy of the as-received specimen displayed a very smooth surface, with few cracks or pores. The post-tensile and post-torsion specimens displayed larger surface cracks, but maintained an overall similar surface finish. The post-three point bend specimen showed no appreciable difference with the as-received wires. These post-specimens displayed the behavior of a material with very little internal stresses. Without these stresses, fewer cracks are formed during the manufacturing process, resulting in fewer stress raisers on the surface. This would explain the smoother surface of these wires after undergoing plastic deformation.

### Comparison of Group I and Group II

The mechanical properties found during the tensile testing are consistent within both groups. There are small standard deviations between groups for E, S_y_ and UTS, as well as no statistically significant differences between the two, showing that there is little variability in the tensile properties of these wires. Some differences between the two did arise during testing. First, since the wires of Group II have a smaller cross-sectional area, they required less axial loading to reach their yield point, as expected. Secondly, the wires of Group II fractured rapidly after reaching their yield strength, resulting in very little plastic deformation; whereas wires of Group I did experience a noticeable amount of permanent deformation. As stated earlier, it is possible that the increase in strain hardening in the manufacture of Group II wires is responsible for making them slightly more brittle than the larger wires of Group I.

The shear modulus found by torsional testing is consistent within each group. Although both wires displayed similar values of G and standard deviations, statistical significance was found between them (P = 0.01). An important difference between the two is the smaller loads required to twist the wires of Group II. The thickness of these wires is 0.002 inches less than that of Group I, which in turn reduces the amount of torque necessary to produce the same angle of twist as in the larger wires.

The three-point bending test best illustrated the difference between the two groups. The flexural rigidity, which is both a material and geometric property, describes the wires resistance to bending in the plane of the thickness. The EI of Group II was approximately 23 percent smaller than that of Group I and strong statistical significance was found (P << 0.05). This demonstrates how the smaller wire, resulting in a smaller moment of inertia, is easier to deflect and thus engage a misaligned bracket. When approximating the cross-sections as perfect rectangles, the wires of Group II have a moment of inertia 28% smaller than that of Group I, which is comparable to the results found.

The results from the mechanical testing indicate that the wires, within their lots, have very consistent mechanical properties along their lengths. Both groups displayed similar values for standard deviation, inter-sample and inter-group. Statistical significance was found for several samples in the torsion and three-point bending tests, showing that some variability is present within groups. As expected, the wires from Group II were easier to axially stretch, twist and bend than the larger wires of Group I. This confirms that, for clinical applications, the larger wires should be used at the beginning of treatment, when severe misalignments require large activation forces, and the smaller wires should be used in the later stages of treatment, where applied forces are less significant.

The surface finish of the as-received wires was very similar for both groups. They displayed a smooth surface, with few cracks and pores, which minimizes friction losses with brackets. However, the post specimens had very different characteristics. The wires of Group I displayed a very rough surface, which differed largely from its as-received state. The wires of Group II displayed a surface that underwent very few changes, conserving an overall smooth surface. As stated earlier, the wires of Group II would require a larger amount of strain hardening during the manufacturing process. It is possible that an extra step was added in the manufacturing of these wires to relieve the internal residual stresses caused by this increase in strain hardening. If an annealing heat treatment was used, the resulting wires would regain some ductility, which would explain the smoother surface of the post specimens.

## CONCLUSION

This work showed that variability does exist in reference to some mechanical properties and physical characteristics between materials, and among samples of the same Groups taken from different lots. Variability between lots could be caused by inconsistent manufacturing methods; this should be taken as a warning to clinicians that variability can occur and influence orthodontic results. Furthermore, from the results from both groups, the testing methods used show high reliability and the parameters selected for evaluation provide all the information that could be necessary in the evaluation and comparison of different wire types in future studies. The testing methods provide the information required by both designers wishing to improve the arch wire properties/characteristics and provide valuable information to clinicians for their practice.

## Figures and Tables

**Fig. (9):**
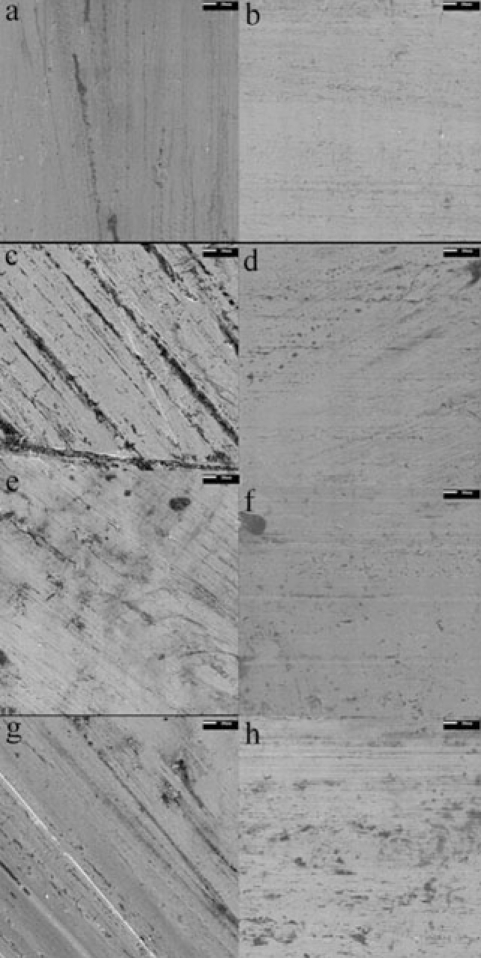
Scanning electron microscopy of surface: 500X magnification, scale bar 10um, Group I; (**a**) as received, (**c**) tensile post-specimen, **(e**) torsion post-specimen, **(g**) Three-Point bending post-specimen; Group II; (**b**) as received, (**d**) tensile post-specimen, (**f**) torsion post-specimen, (**h**) Three-Point bending post-specimen.

**Fig. (10):**
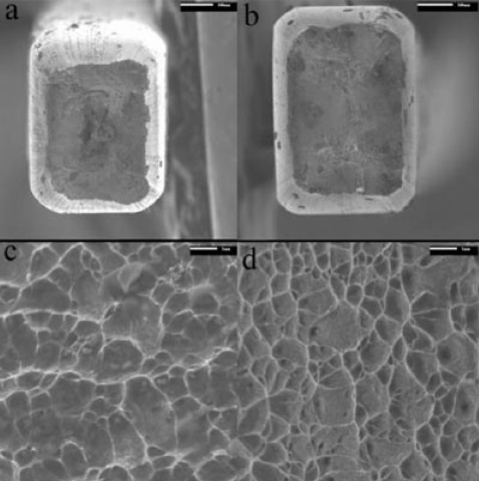
Scanning electron microscopy of fracture surface: Group I: (**a**) 130X, scale bar 100um (**c**) 2000X, scale bar 1um; Group II: (**b**) 130X, scale bar 100um (**d**) 2000X, scale bar 1um.

**Table 1:** Tension Test Results; UTS, YS, E and U_r_ (Mean ± SD); (*) Denotes Statistical Significance (P < 0.05)

Group	n	UTS	Sy	E	U_r_
		MPa (SD)	MPa (SD)	GPa (SD)	MPa (SD)
**I**	10	2041.7 (63.4)	1953.9 (67.0)	169.8 (5.4)	12.99 (1.03)
**II**	10	2098.1 (77.2)	2028.2 (83.0)	168.4 (7.5)	14.60 (0.77)

**Table 2:** Torsion Test Results; G (Mean ± SD); (*) Denotes Statistical Significance (P < 0.05)

Group	Sample	Shear Modulus G	
		[GPa]	
		Intersample	Intergroup (*)
**I**	1	64.2 ± 1.3	63.5 ± 1.8
	2	62.9 ± 0.3	
	3	59.5 ± 1.0 (*)	
	4	57.0 ± 0.7 (*)	
	5	56.4 ± 1.1 (*)	
	6	65.6 ± 2.3	
	7	68.0 ± 3.1 (*)	
	8	69.4 ± 2.3 (*)	
	9	68.3 ± 2.1 (*)	
	10	64.0 ± 6.6	
**II**	1	64.7 ± 0.7 (*)	66.2 ± 2.4
	2	62.7 ± 2.0 (*)	
	3	64.5 ± 3.5	
	4	68.8 ± 1.0 (*)	
	5	62.8 ± 1.2 (*)	
	6	66.7 ± 2.5	
	7	68.1 ± 1.6	
	8	66.8 ± 0.7	
	9	68.3 ± 1.1 (*)	
	10	68.7 ± 1.0 (*)	

**Table 3:** Three-Point Bend Test Results; EI (Mean ± SD); (*) Denotes Statistical Significance (P < 0.05)

Group	Sample	Flexural Rigidity EI	
		[N*mm^2^] x 10^3^	
		Intersample	Intergroup (*)
**I**	1	1.025 ± 0.005 (*)	1.008 ± 0.0021
	2	1.022 ± 0.004 (*)	
	3	1.017 ± 0.009	
	4	0.986 ± 0.013 (*)	
	5	1.026 ± 0.007 (*)	
	6	1.006 ± 0.009	
	7	0.992 ± 0.023	
	8	0.987 ± 0.025	
	9	1.006 ± 0.005	
	10	0.999 ± 0.014	
**II**	1	0.755 ± 0.007 (*)	0.769 ± 0.014
	2	0.765 ± 0.011	
	3	0.787 ± 0.005 (*)	
	4	0.761 ± 0.017	
	5	0.768 ± 0.009	
	6	0.766 ± 0.014	
	7	0.779 ± 0.004 (*)	
	8	0.767 ± 0.016	
	9	0.770 ± 0.019	
	10	0.778 ± 0.004 (*)	

**Table 4:** Cross-Section Area Results (Mean ± SD). ^†^ Not Included in Intergroup Mean and Standard Deviation Calculations for Group II; (*) Denotes Statistical Significance (P < 0.05)

Group	Sample	Area (mm^2^)	
		Intersample	Intergroup (*)
**I**	1	0.291 ± 0.005	0.294 ± 0.004
	2	0.294 ± 0.005	
	3	0.293 ± 0.004	
	4	0.292 ± 0.006	
	5	0.292 ± 0.007	
	6	0.296 ± 0.005	
	7	0.291 ± 0.002	
	8	0.292 ± 0.006	
	9	0.296 ± 0.004	
	10	0.304 ± 0.002 (*)	
**II**	1	0.275 ± 0.003	0.274 ± 0.003
	2	0.269 ± 0.002 (*)	
	3	0.276 ± 0.002	
	4	0.271 ± 0.003 (*)	
	5	0.277 ± 0.001	
	6	0.271 ± 0.002 (*)	
	7^†^	0.302 ± 0.001 (*)	
	8	0.275 ± 0.003	
	9	0.274 ± 0.002	
	10	0.278 ± 0.004	

**Table 5:** Bevel Angle Results (Mean ± SD); (*) Denotes Statistical Significance (P < 0.05)

Group	Sample	Average Bevel Angle (Degrees)	
		Intersample	Intergroup (*)
**I**	1	49.36 ± 2.55	49.17 ± 0.43
	2	49.46 ± 1.44	
	3	49.17 ± 1.93	
	4	49.33 ± 1.25	
	5	49.88 ± 1.09	
	6	49.00 ± 0.88	
	7	49.00 ± 1.49	
	8	48.72 ± 1.04	
	9	49.43 ± 0.81	
	10	48.34 ± 2.04	
**II**	1	49.28 ± 2.80	48.35 ± 1.43
	2	47.79 ± 3.11	
	3	50.40 ± 1.73	
	4	48.75 ± 0.69	
	5	46.62 ± 2.31	
	6	49.91 ± 1.30	
	7	49.43 ± 1.27	
	8	48.11 ± 2.90	
	9	46.58 ± 1.75	
	10	46.60 ± 2.06	

## References

[1] Kapila S, Sachdeva R (1989). “Mechanical properties and clinical applications of orthodontic wires”. Am. J. Orthod. Dentofac.

[2] Santoro M, Nicolay OF, Cangialosi TJ (2001). “Pseudoelasticity and thermoelasticity of nickel-titanium alloys: A clinically oriented review”. Am. J. Orthod. Dentofac.

[3] Krishnan V, Kumar K (2004). “Mechanical Properties and Surface Characteristics of Three Archwire Alloys”. Angle Orthod.

[4] Drake SR, Wayne DM, Powers JM, Asgar K (1982). “Mechanical properties of orthodontic wires in tension, bending and torsion”. Am. J. Orthod.

[5] Kusy RP, Dilley GJ, Whitley JQ “Mechanical properties of stainless steel orthodontic arch wires”. Clinical Materials.

[6] Asgharnia MK, Brantley WA (1986). “Comparison of bending and tension tests for orthodontic wires”. Am. J. Orthod.

[7] Verstrynge A, Humbeeck JV, Willems G (2006). “In-vitro evaluation of the material characteristics of stainless steel and beta-titanium orthodontic wires”. Am. J. Orthod. Dentofac.

[8] Rucker BK, Kusy RP (2002). “Elastic properties of alternative versus single-stranded leveling archwires”. Am. J. Orthod. Dentofac.

[9] Rucker BK, Kusy RP (2002). “Elastic Flexural Properties of Multistranded Stainless Steel Versus Conventional Nickel Titanium Archwires”. Angle Orthod.

[10] Sebanc J, Brantley WA, Pincsak JJ, Conover JP (1984). “Variability of effective root torque as a function of edge bevel on orthodontic arch wires”. Am. J. Orthod.

[11] Steyn CL (1977). “Measurement of edgewise torque force in vitro”. Am. J. Orthod.

[12] Meling TR, Odegaard J, Meling EO (1997). “On mechanical properties of square and rectangular stainless steel wires tested in torsion”. Am. J. Orthod. Dentofac.

[13] American Dental Association/Council on Scientific Affairs (2000). ANSI/ADA Specification No.32-Orthodontic Wires.

[14] Popov EP, Horton Marcia (1998). “Torsion”. Engineering Mechanics of Solids.

[15] Nakano H, Satoh K, Norris R, Jin T, Kamegai T, Ishikawa F, Katsura H (1999). “Mechanical properties of several nickel-titanium alloy wires in three-point bending tests”. Am. J. Orthod. Dentofac.

[16] ASM International handbook committee (1990). Metals Handbook.

